# Hypotonia–cystinuria *2p21* deletion syndrome: Intrafamilial variability of clinical expression

**DOI:** 10.1002/acn3.51464

**Published:** 2021-10-06

**Authors:** Atif Towheed, Christian L. Hietanen, Vasudeva G. Kamath, Larry N. Singh, Angela Ho, Kristin Engelstad, Kayla Cornett, Jacqueline Montes, Darryl De Vivo

**Affiliations:** ^1^ Touro College of Osteopathic Medicine Middletown NY 10940; ^2^ Children’s Hospital of Philadelphia Research Institute Philadelphia PA 19104; ^3^ Department of Neurology Columbia University Irving Medical Center New York NY 10032; ^4^ Department of Rehabilitation and Regenerative Medicine (Physical Therapy) Columbia University Irving Medical Center New York NY 10032; ^5^ Department of Neurology and Pediatrics Columbia University Irving Medical Center New York NY 10032

## Abstract

Two siblings presented similarly with congenital hypotonia, lactic acidosis, and failure to thrive. Later in childhood, the brother developed cystinuria and nephrolithiasis whereas the older sister suffered from cystinuria and chronic neurobehavioral disturbances. Biopsied muscle studies demonstrated deficient cytochrome c oxidase activities consistent with a mitochondrial disease. Whole exome sequencing (WES), however, revealed a homozygous *2p21* deletion involving two contiquous genes, *SLC3A1 (deletion of exons 2‐10)* and *PREPL (deletion of exons 2‐14)*. The molecular findings were consistent with the hypotonia–cystinuria *2p21* deletion syndrome, presenting similarly in infancy with mitochondrial dysfunction but diverging later in childhood and displaying intrafamilial phenotypic variability.

## Introduction

**Figure 1:**
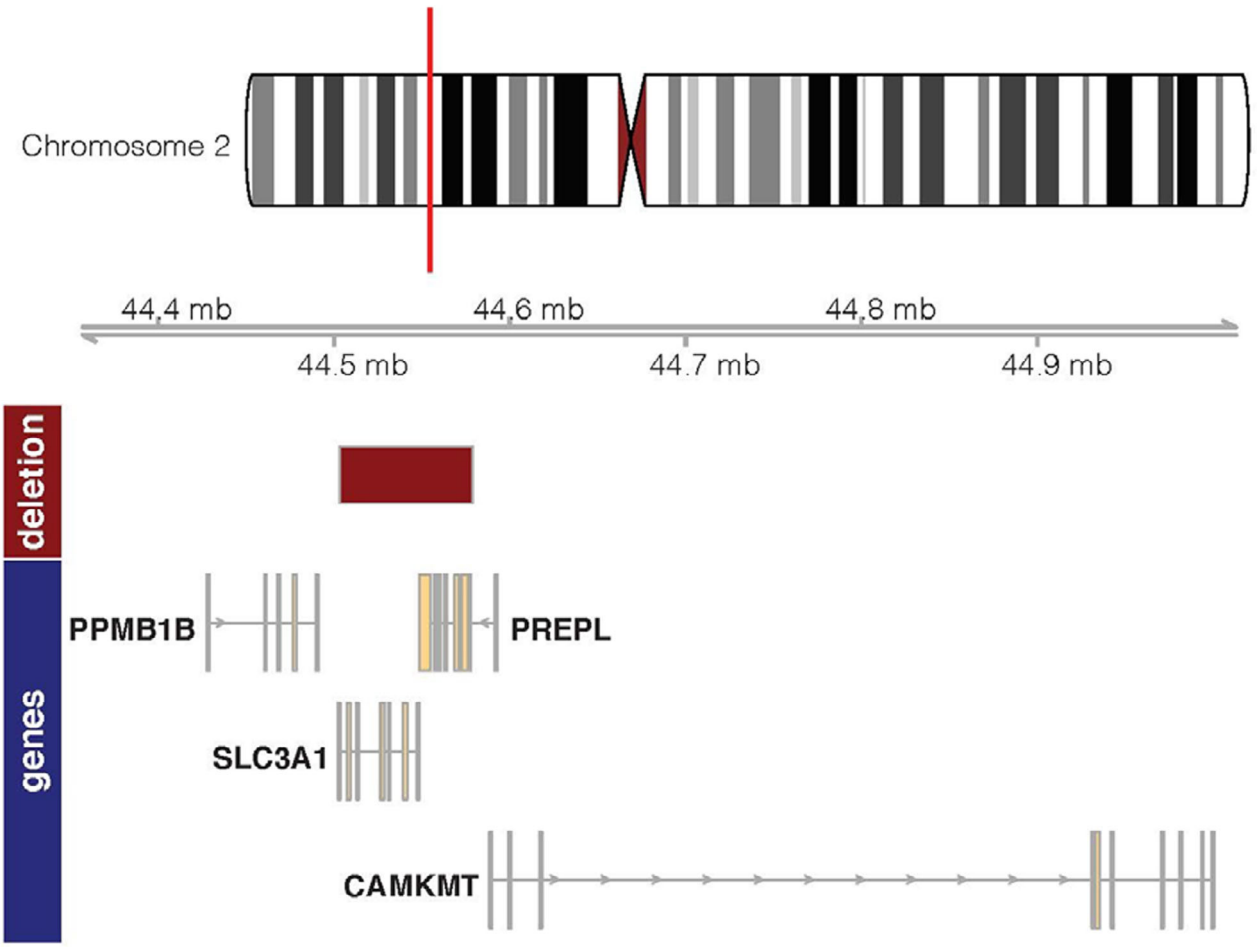
Genomic organization of *SLC3A1* and *PREPL* and its neighboring genes on *Chromosome 2*. Note: Gviz software was used for visualization of this genomic region.^24^

Homozygous deletion of chromosome *2p21,* containing two closely located genes, *SLC3A1,* and *PREPL* (Fig. 1), has been linked to the hypotonia–cystinuria syndrome (HCS; OMIM #606407).^1^ Symptoms include generalized hypotonia at birth, failure to thrive, cystinuria, and growth retardation. The deletion size typically ranges from 23.8 to 75.5 kb. Taroni (2019) reported that the HCS is a rare disease with only 26 cases reported to date resulting from eight different deletions.^2^ From the published reports, HCS is not usually associated with mitochondrial dysfunction. Literature search yielded two previous reports of HCS with mitochondrial dysfunction (Parvari,^3^ 2001 and Zaffanello,^4^ 2003).

*SLC3A1* encodes the heavy chain subunit of the cystine and dibasic amino acid transporters in the renal proximal tubule and small intestine. Inactivation causes cystinuria type I (OMIM 220100).^5^, ^6^, ^7^ The *SLC3A1* gene spans 46 kb and has 10 exons.^8^, ^9^ Cystinuria is characterized by a defect in resorption of cystine, ornithine, lysine, and arginine. High concentrations of cystine in the distal tubule results in the precipitation of cystine stones.

*PREPL* is a prolyl oligopeptidase‐like protein homologous to the prolyl oligopeptidase family.^10^ All proteins in this family have been implicated in various clinical conditions; prolyl endopeptidase (PREP; OMIM 600400), oligopeptidase B (OpdB) (role in host cell invasion by *Trypanosoma cruzi*, Chagas disease),^11^ acylaminoacyl‐peptidase (APEH; OMIM 102645), and dipeptidase IV (DPPIV; OMIM 102720). Isolated loss of *PREPL* gene has been implicated in congenital myasthenic syndrome‐22 (CMS22; OMIM 616224).^12^

We report two siblings with a similar early clinical presentation consistent with mitochondrial disease. Muscle biopsy revealed partial cytochrome c oxidase deficiency. The later clinical courses in the two siblings diverged with severe nephrolithiasis dominating the one case and neurobehavioral disturbances dominating the other. We offer some speculations regarding the intrafamilial variability of the clinical presentations during later development.

## Patients and Methods

### Clinical presentation

#### Patient 1

Patient 1, a 19‐year‐old male with a history of failure to thrive, hypotonia, developmental delay, and severe gastroesophageal reflux, required a gastrostomy in infancy. Additional findings included lactic acidosis, mild dysmorphism of facial features, a nasal speech pattern consistent with velopharyngeal insufficiency and color blindness.

At age 7 years, a chest x‐ray incidentally revealed kidney stones prompting further investigation. Twenty‐four‐hour urine evaluation confirmed high cystine excretion. The patient later required percutaneous nephrolithotomy for removal of 19 stones (100% cystine composition) leading to a diagnosis of cystinuria and urolithiasis. Poor renal function prompted aggressive treatment with Polycitra‐K and Sulfa prophylaxis. A muscle biopsy confirmed partial cytochrome c oxidase deficiency (40.61% of control).

Generalized muscle weakness and exertional fatigue became evident with increasing physical demands requiring wheelchair for more sustained activities. Growth hormone (GH) therapy was started at age 12 years when he was below the 5th percentile for height. Five years later height was at the 17th percentile. Academic and social concerns worsened during higher education. At age 14 years, he was diagnosed with mild autism spectrum disorder (ASD). Currently, he is doing well academically and anticipating entering college shortly.

Genetic analysis using WES for Patient 1 revealed nuclear mutations in *Chr2:44503092‐44578898del (*homozygous*) (SLC3A1:E2_10del/* PREPL:E2_14del homozygous), *TTN c.59344A>C (p.K19782Q) and c.50026A>C (p.T16676P)* in trans, and *OR5H14 c.4G>A (p.E3K)* homozygous. Mitochondrial sequencing revealed *MTRNR1 m.884T>C* 2.1% heteroplasmy VUS but was negative for large deletions and other known pathogenic mutations.

**Figure 2:**
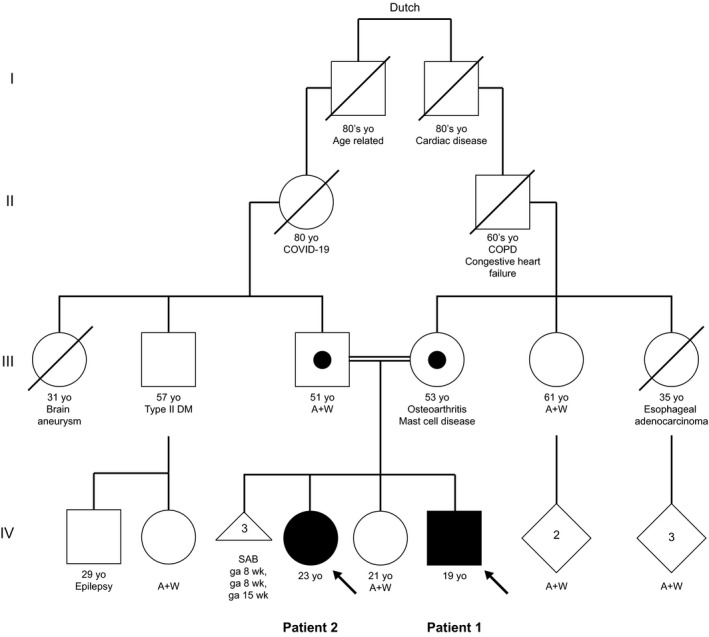
Family pedigree.

The family history is remarkable with his sister (Patient 2) initially presenting with similar symptoms. Both maternal and paternal family members are of Dutch descent (Fig. 2). WES analysis confirmed the parents as heterozygous carriers for the *2p21* microdeletion consistent with an autosomal recessive pattern of inheritance.

#### Patient 2

Patient 2 is a 23‐year‐old female with a history of hypotonia, lactic acidosis, feeding, and gastrointestinal motility problems consistent with congenital onset mitochondrial disease.

She suffered developmental delays in early childhood with generalized muscle weakness, mild gait disturbance, exercise intolerance, fatigue, and nasal voice. The patient later demonstrated serious neurobehavioral disturbances and learning difficulties. She was diagnosed with ASD, complicated by comorbid features of attention deficit hyperactivity disorder, obsessive‐compulsive disorder, and chronic anxiety. The medications included lisdexamfetamine, fluoxetine, omeprazole, l‐thyroxine, vitamin B12, coenzyme Q10, biotin, and GH.

In contrast to her brother, Patient 2 developed cystinuria much later and remains free of renal calculi. Muscle biopsy revealed 29% cytochrome c oxidase activity compared to the control. G‐banding karyotype analysis proved unremarkable likely due to poor resolution.

WES analysis revealed the same microdeletion found in her brother. In addition, she had distinct nuclear mutations involving *GRIN3A c*.*2309G>A (p.G770D) and c.3332G>A (p.R1111Q) (*heterozygous*) and USP25 c.2929A>C (p.K977Q) (*homozygous). Other than a benign polymorphism in mt tRNA‐*Lys m.8251G>A*, mitochondrial genome analysis was unremarkable.

**Figure 3:**
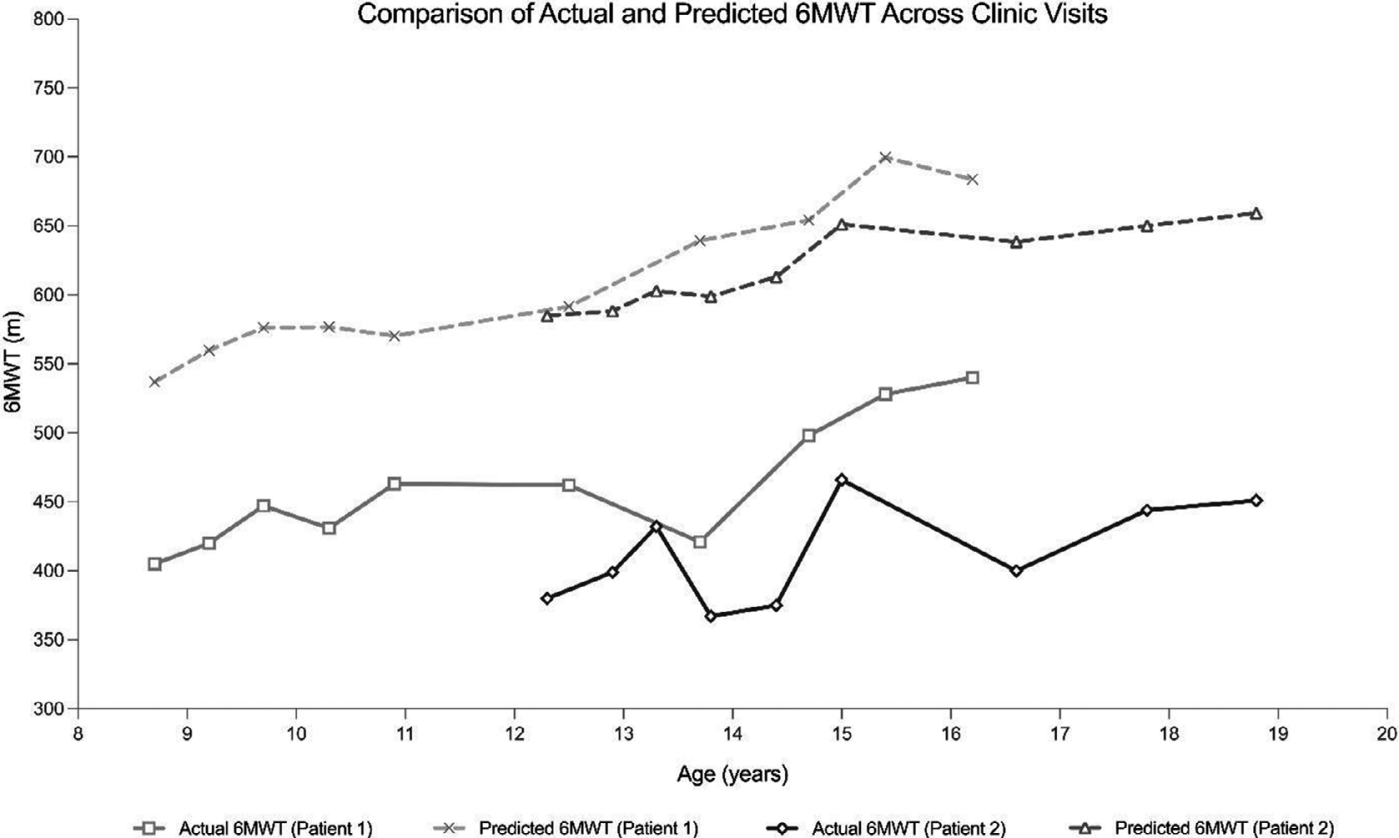
Comparison of actual versus predicted 6MWT distance across clinic visits.

Both patients were evaluated biannually for 7 years and the 6‐minute walk test (6MWT) was used to monitor ambulatory function and endurance.^13^ Distance walked continued to improve, but both patients consistently underperformed walking approximately 150 meters less on average than predicted (Fig. 3). Neither patient displayed laboratory evidence of fatigue. Patient 1 had a normal average fatigue index of −2.4% over ten 6MWTs. Patient 2 also had a normal average fatigue index of 2.4% over nine 6MWTs. Additional exercise testing performed by Patient 1 at ages 15 and 16 years, revealed aerobic capacity that was more than 50% below average (VO_2_ peak = 20.1 and 22.0 mL/kg/min, respectively) and diminished local muscle oxygen uptake during exercise using near infrared spectroscopy (NIRS).^14^, ^15^ NIRS is a noninvasive method to determine oxygen saturation in tissues which quantifies muscle oxygen uptake and oxidative capacity from an exercise tolerance test.

## Discussion

The patients presented here have homozygous deletions of *SLC3A1* and *PREPL* which according to Chabrol et al. and Barthholdi et al. would be classified as HCS. Chabrol et al. reported respiratory chain deficiency observed in all patients with *2p21* deletion syndrome patients.^16^, ^17^ Remarkably, the above clinical presentations are inconsistent with this report of HCS as the clinical evidence for a mitochondrial disorder in HCS is uncommon.^2^

In contrast, the presence of mitochondrial disease is relatively common in “*2p21* microdeletion syndrome” which involves the deletion of at least four contiguous genes on chromosome 2, *SLC3A1*, *PREPL*, *PPM1B*, and *CAMKMT*. *PPM1B*, and *CAMKMGT* mutations were not found in these patients.^18^ The two siblings were initially diagnosed with partial cytochrome c oxidase deficiency. Subsequent WES and cSNP‐array confirmed the homozygous microdeletion of *SLC3A1* and *PREPL*. Therefore, these findings confirmed the diagnosis of HCS presenting atypically with mitochondrial dysfunction and partial cytochrome c oxidase deficiency.

Mitochondrial dysfunction in the setting of *SLC3A1 and PREPL* mutations is intriguing but not easily explained. Neither gene, has been shown to play a direct role in mitochondrial function. We do wonder if mitochondrial dysfunction could be the result of a blunted response to oxidative stress. Reduced *SLC3A1* expression may affect glutathione (GSH) synthesis as cysteine serves as the rate‐limiting precursor for GSH synthesis. Dysregulation of GSH could contribute to increased cellular vulnerability to oxidative stress.^19^ Further studies will be necessary to pursue this speculation.

HCS syndrome is generally accompanied by GH deficiency. According to the current understanding of familial isolated GH deficiencies, there are at least four possibilities.^20^ However, none of these possibilities explain the GH deficiency in this clinical setting.

Although the two patients have the same microdeletion, their clinical presentation later diverged significantly. These differences could be attributed, at least in part, to the additional mutations in the respective genetic backgrounds. For example, Patient 2 has a mutation in *GRIN3A* which could potentially impact synaptic elements in the brain contributing to severe neurobehavioral issues.

The absence of renal stones and late presentation of cystinuria in Patient 2 also is perplexing. We would expect *Patient 2* to present with urolithiasis and cystinuria earlier. The difference in clinical symptoms of urolithiasis in Patient 1 versus Patient 2 despite sharing the same deletions supports the observation of intrafamilial phenotypic variability; but the mechanism underlying this variability remains unexplained. According to Lahme et al., mutation of the *SLC3A1* gene was detected in only 50% of patients presenting with cystinuria suggesting multiple factors contributing to the phenotype.^21^ However, according to Parvari et al. a low rate of *SLC3A1* mutation in cystinuria likely was due to lack of screening for alternative isoform sequences that play a role in the renal transport system.^22^ Detailed molecular studies are therefore required to further characterize the underlying mechanisms.

Gender could potentially play a role in the differences observed between the two siblings. According to a report by Nagamori et al., heterodimeric partners of basic amino acid transporters in the proximal kidney tubule were found to be expressed in a pattern determined by the biological sex.^23^ This study suggests a modulating role of estrogen on the tubular amino acid transport.

In summary, HCS is a complex genetic syndrome complicated by the variable presentation of clinical symptoms in early life and kidney dysfunction with urolithiasis later in life. The mechanism underlying mitochondrial dysfunction remains obscure. We speculate that oxidative stress may contribute but direct measures of GSH will be necessary to support this speculation. Management of GH deficiency clearly is indicated but, again, the mechanism underlying this endocrine disturbance needs to be elucidated.

## Conflict of Interest

The authors are not aware of any conflict of interest involved in this report.

## Funding Information

No funding information provided.
